# Endothelial-Derived Oxidative Stress Drives Myofibroblastic Activation and Calcification of the Aortic Valve

**DOI:** 10.1371/journal.pone.0123257

**Published:** 2015-04-13

**Authors:** Emily J. Farrar, Geoffrey D. Huntley, Jonathan Butcher

**Affiliations:** 1 Department of Biomedical Engineering, Cornell University, Ithaca, New York, United States of America; 2 Mayo Medical School, Mayo Clinic, Rochester, Minnesota, United States of America; Brigham and Women's Hospital, Harvard Medical School, UNITED STATES

## Abstract

**Aims:**

Oxidative stress is present in and contributes to calcification of the aortic valve, but the driving factors behind the initiation of valve oxidative stress are not well understood. We tested whether the valve endothelium acts as an initiator and propagator of oxidative stress in aortic valve disease.

**Methods and Results:**

Calcified human aortic valves showed side-specific elevation of superoxide in the endothelium, co-localized with high VCAM1 expression, linking oxidative stress, inflammation, and valve degeneration. Treatment with inflammatory cytokine TNFα increased superoxide and oxidative stress and decreased eNOS and VE-cadherin acutely over 48 hours in aortic valve endothelial cells (VEC) and chronically over 21 days in *ex vivo* AV leaflets. Co-treatment of VEC with tetrahydrobiopterin (BH_4_) but not apocynin mitigated TNFα-driven VEC oxidative stress. Co-treatment of *ex vivo* AV leaflets with TNFα+BH_4_ or TNFα+peg-SOD rescued endothelial function and mitigated inflammatory responses. Both BH_4_ and peg-SOD rescued valve leaflets from the pro-osteogenic effects of TNFα treatment, but only peg-SOD was able to mitigate the fibrogenic effects, including increased collagen and αSMA expression.

**Conclusions:**

Aortic valve endothelial cells are a novel source of oxidative stress in aortic valve disease. TNFα-driven VEC oxidative stress causes loss of endothelial protective function, chronic inflammation, and fibrogenic and osteogenic activation, mitigated differentially by BH_4_ and peg-SOD. These mechanisms identify new targets for tailored antioxidant therapy focused on mitigation of oxidative stress and restoration of endothelial protection.

## Introduction

Aortic valve disease (AVD) causes approximately 15,000 deaths per year in the United States, occurring in 2.8% of Americans over the age of 75 [1]. AVD is an active process driven by complex intercellular interactions [2] that is pathobiologically unique from other cardiovascular diseases [3–5]. Valve endothelial cells (VEC), which line the surface of the valve, are phenotypically different from other endothelial cell populations [6–9] and must be investigated for unique pathological mechanisms. VEC are known to play a unique role in regulating the properties of valve tissue in response to the dynamic cardiac environment [10–11], but their role in early disease is not known. Disruption of VEC function is an early and persistent feature of AVD [12–14], but it is not known if disrupted VEC actively contribute to valve calcification. Development of treatments that would protect against AVD by maintaining VEC protective function and blocking potential active contributions to calcification are hampered by a lack of understanding of the underlying mechanisms of VEC pathology.

VEC production of nitric oxide (NO) is protective against AVD, but is reduced in calcified valves [15–18]. In vascular disease, NO is reduced due to a phenomenon known as eNOS uncoupling [19], however, it is not known if eNOS uncoupling represents a significant source of oxidative stress in AVD. Uncoupled eNOS produces superoxide rather than nitric oxide due to a lack of the eNOS co-factor tetrahydrobiopterin (BH_4_) [20]. Increased superoxide reacts with NO to form peroxynitrite (ONOO^-^), further reducing the bioavailability of NO [21], or excess superoxide is dismutated to hydrogen peroxide (H_2_O_2_) [22]. Superoxide and hydrogen peroxide are increased in calcifying regions of the aortic valve [23–24] and contribute to osteogenic activation of valve interstitial cells [25]. If eNOS uncoupling is a significant phenomenon in the valve endothelium, VEC-derived oxidative stress could be an important and accessible target in the development of treatments to prevent valve calcification.

Inflammation has long been clinically linked to oxidative stress [26] and eNOS uncoupling [27] in vascular endothelial cells, but it is not known whether a similar link exists in valve endothelial cells as a mechanism of valvular disease. Inflammatory activation of the endothelium correlates proportionally with calcification in AVD [28]. Inflammation induces early and sustained expression of endothelial inflammatory adhesion molecules VCAM-1 and ICAM-1 [29]. Inflammatory cytokine TNFα has been identified as a key effector of pro-inflammatory signaling in valve endothelial cells and of pro-calcific signaling in valve interstitial cells [30–32]. TNFα can also initiate a pro-disease endothelial to mesenchymal transition in adult VEC [33–34]. However, it is not known if TNFα causes VEC to actively contribute pro-calcific oxidative stress to the diseased valve environment.

In this study, we establish that inflammatory cytokine TNFα drives increased oxidative stress in the valve endothelium, identifying valve endothelial cells as a novel source of elevated oxidative stress in AVD. We show that TNFα-driven endothelial oxidative stress, in part from eNOS uncoupling, causes endothelial dysfunction and exacerbates endothelial inflammatory response and that these effects can be prevented by treatment with TNFα+tetrahydrobiopterin (BH_4_) or TNFα+peg-SOD. Results from our *ex vivo* AV leaflet culture show that blocking endothelial oxidative stress also mitigates TNFα-driven myofibroblastic activation, extracellular matrix remodeling, and calcification in the valve tissue. These findings identify a novel pathophysiological mechanism by which TNFα-driven endothelial oxidative stress initiates downstream calcification of the aortic valve. Antioxidant therapies that protect against endothelial-derived increases in oxidative stress can mitigate valve disease progression by preserving the protective capabilities of the endothelium.

## Methods

### Human aortic valves

Calcified human aortic valves were obtained from adults undergoing planned, non-elective valve replacement surgery at Robert Packer Hospital in Sayre, PA. The Guthrie Institutional Review Board at Robert Packer Hospital and the Institutional Review Board for Human Participants at Cornell University approved all procedures (IRB#0908–24, “Gene expression and phenotypic changes in stenotic aortic valves”). Written informed consent was obtained from all participants. The investigation conformed to the principles outlined in the Declaration of Helsinki. Patient age range was 65–90 years, with a mean age of 76.2 years, 21 samples total. Dihydroethidium (DHE) superoxide detection and immunofluorescence were performed as described in the S1 Supplement.

### Aortic valve endothelial cells

Valve endothelial cells (VEC) were harvested from porcine aortic valves (Shirk Meats, Dundee, NY). Use of porcine cells, a widely-accepted human valve analog, provided a large, controlled population of VEC that were screened for phenotype and purity using q-rtPCR and immunofluorescence for CD31, eNOS, VE-cadherin, and absence of αSMA (see S1 Supplement). VEC were found to maintain their endothelial phenotype and purity out to passage six, thus all experiments were conducted between P4 and P6. In all *in vitro* experiments, VEC were cultured at 100,000 cells/cm^2^ on 3-D collagen hydrogels recapitulating the native valve environment, as described previously [35].

### Endothelial oxidative stress state assessment

Human recombinant TNFα was administered at dosages from 10–100ng/mL and determined to have a significant inflammatory effect (VCAM-1 upregulation, NFκB nuclear translocation) with no statistically significant VEC apoptosis at 30ng/mL, as previously shown [33]. H_2_O_2_ dosage (1 μmol/L) was chosen to recapitulate peak increases in secreted H_2_O_2_ by VEC stimulated with 30ng/mL TNFα. Acute increases in oxidative stress due to TNFα were measured across 0–4 hours. Peak effect occurred and is presented here at 30 minutes post-treatment. Intracellular oxidative stress was assessed via DHE fluorescence and CM-H2DCF-DA fluorescence, quantified on a microplate reader and with confocal microscopy. Secreted NO was measured using the Griess assay. Secreted H_2_O_2_ was measured by the Fluoro H_2_O_2_ assay. Mitochondrial reactive oxygen species were measured using MitoSOX Red assay, quantified on a microplate reader and with confocal microscopy. Efficacy of antioxidant administration was optimized for pre-treatment time and dosage. Peak effect was achieved by administering 500μmol/L L-NAME 30 minutes prior to TNFα treatment and all other co-treatments concurrently with TNFα, using 10 μmol/L BH_4_, 100 μmol/L apocynin (acetovanillone), and 20U/mL Cu-Zn-Superoxide dismutase-polyethylene glycol (peg-SOD).

### Ex vivo aortic valve leaflets

Porcine aortic valve leaflets were harvested sterilely from fresh porcine hearts (Shirk Meats, Dundee, NY). Leaflets were immediately rinsed in sterile PBS, transported on ice, and transferred to Dulbecco’s Modified Eagle Medium within two hours. D-MEM was supplemented with 10% fetal bovine serum and 1% penicillin-streptomycin, adjusted to a pH of 7.2. 30ng/mL of TNFα was added directly to the media, supplemented with 10 μmol/L BH_4_ or 20U/mL peg-SOD, where noted. This culture media was changed every 48 hours for 21 days. TUNEL assay for apoptosis was performed to confirm cell viability and DHE or MitoSOX Red assay were used to detect oxidative stress. Russell-Movat pentachrome staining was used to visualize extracellular matrix components and Alizarin Red S in combination with von Kossa staining was used to assess calcification. Western blot, mRNA isolation, q-rtPCR, and immunofluorescence were performed according to standard practices, as described in the S1 Supplement.

### Statistical Analysis

Data is expressed as mean +/- standard error of the mean (SEM). All comparisons between two groups were made using two-tailed, unpaired t-tests assuming unequal variance. Comparisons between multiple groups were made using one-way ANOVA with Tukey’s post hoc tests. Differences between means were considered significant when p < 0.05.

## Results

### Calcified human aortic valves show elevated endothelial superoxide in a side specific manner, co-localized with inflammatory activation

**Fig 1:**
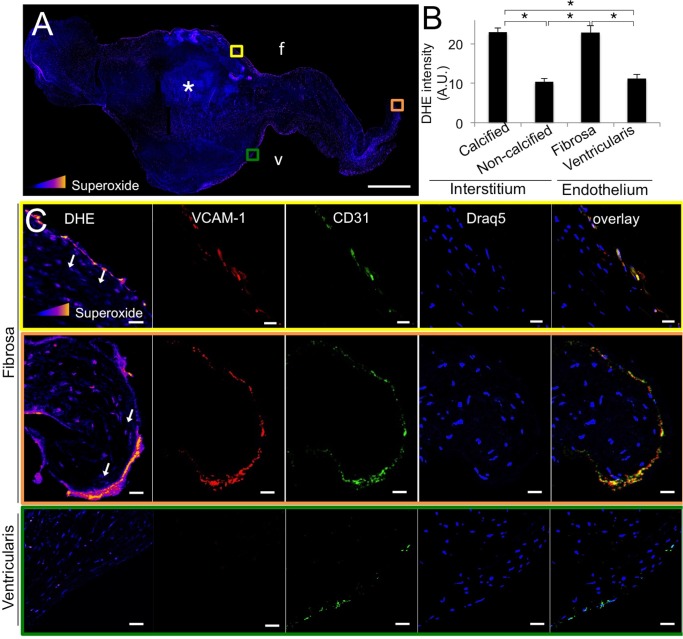
Superoxide is elevated in the endothelium of calcified human aortic valves. **A,** Superoxide staining (DHE) of calcified human aortic valve leaflets. Asterisks indicate calcific nodule. Intensity of superoxide staining is colorimetrically scaled, with yellow indicating most intense (inset triangle). Colored boxes indicate region magnified in lower panels; f indicates fibrosa, v indicates ventricularis. Scale bar = 1 mm. **B**, Quantification of DHE intensity (superoxide) across all valve samples, presented as DHE intensity in different regions of calcified valves (cHAV), n = 21. * indicates p < 0.05 between indicated groups. **C**, Co-localization of elevated superoxide with VCAM-1 and CD31 expression in calcified human aortic valve leaflet endothelium. Representative images from n = 21 valves. Scale bar = 20μm.

We first examined superoxide levels in calcified (cHAV) human aortic valves, using DHE intensity. Superoxide levels were significantly increased in calcified regions of the valves (Fig 1A) and in the fibrosa endothelium, but not in non-calcified regions or the ventricularis endothelium (Fig 1A and 1B). Elevated endothelial superoxide was associated with the fibrosa side of the stenotic human aortic valve, where calcific lesions are known to form. Across cHAV samples, regions of the endothelium with high levels of superoxide were positive for both VCAM-1 and CD31 (Fig 1C), indicating a link between inflammatory endothelial activation and elevated endothelial superoxide. The interstitial cells directly beneath the human VEC (hVEC) did not have significantly increased superoxide (arrows). hVEC on the ventricularis side were positive for CD31, but did not have significantly increased superoxide or elevated VCAM-1 expression (Fig 1C).

### Side-specific expression of endothelial superoxide dismutase in calcified human aortic valves

**Fig 2:**
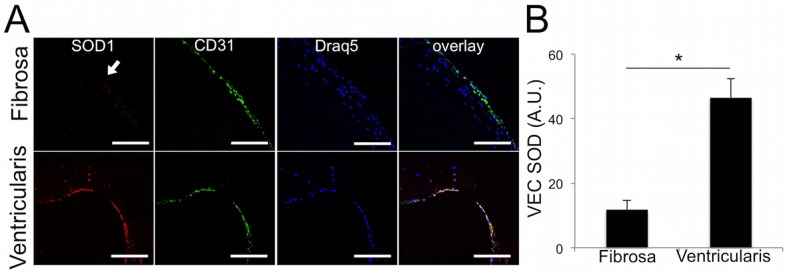
Calcified human aortic valves have lower expression of SOD on the fibrosa endothelium than on the ventricularis. A. Immunofluorescence for endothelial protein CD31 and SOD1 revealed little to no expression of SOD1 (arrow) on the fibrosa endothelium of calcified aortic valve leaflets. The ventricularis showed strong SOD1 expression in the endothelium. **B**, Quantification of the pixel intensity of SOD1 in the CD31+ endothelial cells in cHAV, n = 21. The fibrosa endothelium had significantly less SOD1 than the ventricularis in cHAV. * indicates p < 0.05 between groups, Student’s t-test.

The fibrosa endothelium of diseased valves showed little to no SOD1 expression, while the ventricularis endothelium retained strong expression of SOD1 (Fig 2A). Quantitative analysis of SOD1 expression in the CD31^+^ endothelial cells of cHAV confirmed that the fibrosa endothelium showed significantly less SOD1 than the ventricularis endothelium (Fig 2B).

### TNFα causes increased oxidative stress and decreased NO in VEC

**Fig 3:**
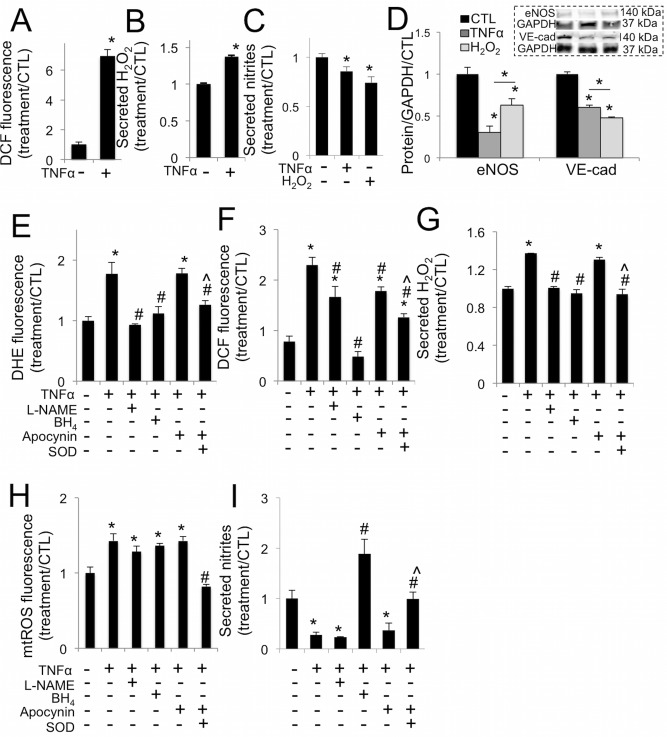
TNFα drives increased oxidative stress in aortic valve endothelial cells via eNOS uncoupling. **A**, TNFα increases oxidative stress in VEC at 30 minutes. **B**, TNFα increases hydrogen peroxide (H_2_O_2_) secretion from VEC at 30 minutes. **C**, TNFα or H_2_O_2_ decrease nitric oxide secretion from VEC at 48 hours (n = 4). **D**, TNFα or H_2_O_2_ decrease eNOS and VE-cadherin expression in VEC at 48 hours. Representative western blot images (inset) and blot quantification. **E,** L-NAME, BH_4_, or peg-SOD but not apocynin block increases in superoxide (DHE) in VEC caused by TNFα, at 30 minutes. **F**, L-NAME, apocynin, and peg-SOD mitigate increases in general oxidative stress (DCF) caused by TNFα at 30 minutes, but only BH_4_ completely blocks superoxide increase, maintaining control levels. **G**, L-NAME, BH_4_, or peg-SOD but not apocynin block increases in H_2_O_2_ secreted by VEC at 30 minutes caused by TNFα at 30 minutes. **H**, TNFα drives increased mtROS, mitigated only by co-treatment with SOD. **I**, BH_4_, or peg-SOD but not L-NAME or apocynin block decreases in nitric oxide secretion in VEC caused by TNFα at 48 hours. * indicates p < 0.05 versus control. # indicates p < 0.05 versus TNFα. ^ indicates p < 0.05 versus apocynin. N = 4.

TNFα caused a significant increase in VEC intracellular oxidative stress (Fig 3A), superoxide (S1 Fig), and secreted H_2_O_2_ (Fig 3B), 30 minutes after treatment. After 48 hours, TNFα or H_2_O_2_ treatment decreased VEC secretion of nitric oxide (Fig 3C) and decreased expression of eNOS and VE-cadherin proteins (Fig 3D and S2 Fig).

To elucidate the source of increases in endothelial oxidative stress and the resulting endothelial dysfunction, we administered NOS inhibitor L-NAME, eNOS co-factor BH_4_, NADPH oxidases inhibitor apocynin, or peg-SOD in conjunction with TNFα treatment. At 30 minutes, L-NAME, BH_4_, and peg-SOD blocked TNFα-stimulated increases in VEC superoxide, but apocynin did not (Fig 3E). L-NAME and apocynin reduced overall oxidative stress compared to TNFα alone, and peg-SOD lowered oxidative stress further still. Only eNOS co-factor BH_4_ restored VEC oxidative stress back to control levels (Fig 3F). Similar trends were observed in H_2_O_2_ secretion: co-administration of L-NAME, BH_4_, or peg-SOD blocked increases in secreted H_2_O_2_ by VEC, but apocynin did not (Fig 3G). TNFα similarly drove increases in VEC mitochondrial reactive oxygen species (mtROS), which were only abrogated by peg-SOD (Fig 3H). Over 48 hours, only BH_4_ or peg-SOD maintained control levels of nitric oxide secretion (Fig 3I). Catalase co-treatment was able to reduce general oxidative stress state in VEC, but was found to cause a significant increase in apoptosis, loss of VE-cadherin, nuclear translocation of NFκB, and VCAM-1 expression (S3 Fig). Thus, catalase treatment had off-target effects on VEC that were significant enough to prevent further investigation of its viability as a pro-endothelial valve treatment against inflammation and calcification.

### Mitigating the effects of TNFα-oxidative stress rescues endothelial function and abates VEC inflammatory response

**Fig 4:**
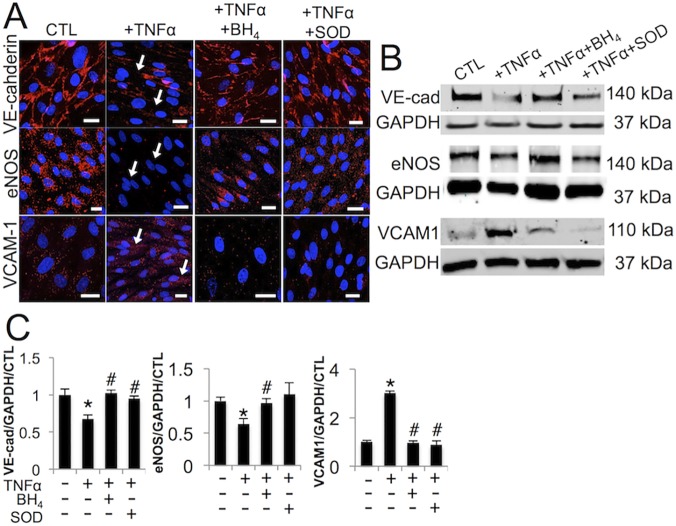
Blocking eNOS uncoupling mitigates TNFα-induced endothelial dysfunction and inflammatory response. **A,** TNFα decreases VE-cadherin and eNOS expression in VEC over 48 hours (arrows), rescued by BH_4_ or peg-SOD. TNFα increases VCAM-1 expression in VEC over 48 hours, mitigated by BH_4_ or peg-SOD. **B**, Western blot analysis of VE-cadherin (VE-cad), eNOS, and VCAM-1 protein in same conditions as A, with GAPDH housekeeping protein. **C**, Quantification of western blot results, normalized to GAPDH and control, n = 3. * indicates p < 0.05 versus control, # indicates p < 0.05 versus TNFα. Scale bar = 20μm. Images representative of three independent experiments.

We then examined the protein-level effects of BH_4_ and peg-SOD on VEC when applied in conjunction with TNFα treatment. TNFα decreased VE-cadherin and eNOS and increased VCAM-1 protein expression in VEC (Fig 4A, arrows). Co-treatment of VEC with BH_4_ or peg-SOD protected against loss of VE-cadherin and eNOS and reduced VCAM-1 response to TNFα. Western blot analysis confirmed this result (Fig 4B and 4C and S4 Fig).

### TNFα increases VEC oxidative stress in *ex vivo* aortic valve leaflets, rescued by antioxidants

**Fig 5:**
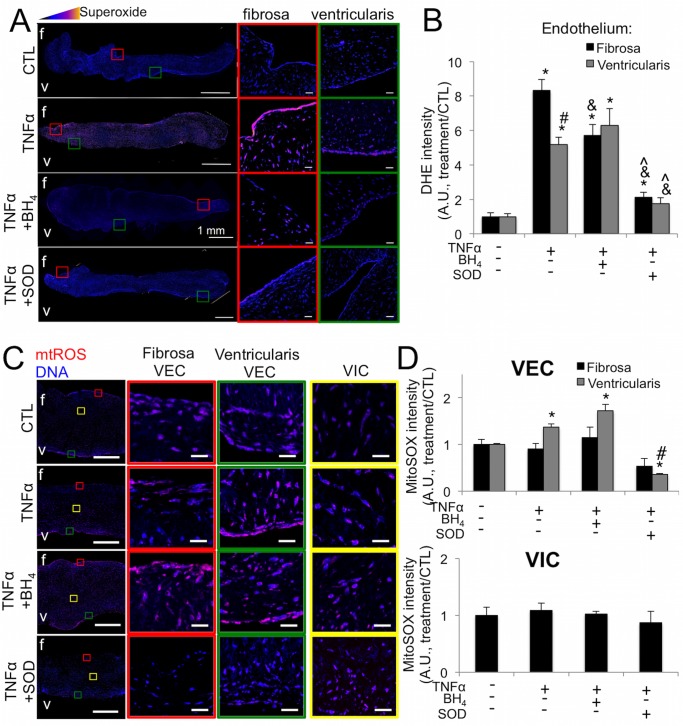
TNFα induces side-specific endothelial oxidative stress in *ex vivo* aortic valve leaflets. **A**, TNFα causes increased superoxide in the fibrosa endothelium of *ex vivo* porcine aortic valve leaflets cultured for 21 days, revealed by DHE staining. Superoxide is mitigated on the fibrosa by co-treatment with BH_4_ and on both the fibrosa and ventricularis by peg-SOD. Pixel intensity of superoxide levels is scaled colorimetrically (inset triangle) to show regions of highest superoxide. Colored boxes indicate regions magnified on right, showing endothelium on fibrosa and ventricularis sides of valve. Scale bar is 1mm in left images, 20μm in magnified images. **B**, Quantification of endothelial superoxide in *ex vivo* aortic valves stained with DHE reveals side-specific rescue-effects of BH_4_ and more pronounced mitigation of superoxide on both sides of the valve by peg-SOD. **C**, TNFα increases mtROS in the ventricularis VEC of *ex vivo* AV leaflets. **D**, Quantification of mtROS fluorescenc. Ventricularis-specific increases in mtROS are mitigated by peg-SOD but not BH_4_. * indicates p < 0.05 versus control condition. # indicates p < 0.05 versus fibrosa endothelium in same condition. & indicates p < 0.05 versus same side endothelium in TNFα condition. ^ indicates p < 0.05 versus same side endothelium in TNFα+BH_4_. N = 6.

We then used *ex vivo* culture of healthy porcine AV leaflets to examine tissue-level effects of TNFα-driven endothelial oxidative stress and the ability of BH_4_ or peg-SOD to mitigate TNFα effects. No significant apoptosis was observed in the leaflets throughout the course of the experiment (S5 Fig). Similar to our findings *in vitro*, over 21 days TNFα increased endothelial superoxide, most distinctly in the fibrosa endothelium (Fig 5A). BH_4_ treatment mitigated fibrosa but not ventricularis-side increases in superoxide. Peg-SOD lowered endothelial superoxide even further than BH_4_, significantly decreasing levels on both the fibrosa and ventricularis sides (Fig 5B). To elucidate a potential mechanism behind differential BH_4_ and peg-SOD effects, we examined the contribution of mitochondrial reactive oxygen species (mtROS) within our *ex vivo* leaflets. We found that TNFα indeed increased levels of mtROS, specifically within the ventricularis endothelium (Fig 5C). Quantification of mtROS fluorescence revealed a 42±9.3% increase in mtROS only in ventricularis VEC and not in fibrosa VEC or VIC, which was mitigated by co-treatment with SOD, but not BH_4_ (Fig 5D).

### Antioxidants improve endothelial function and repress valve interstitial cell myofibroblastic activation in aortic valve leaflets

**Fig 6:**
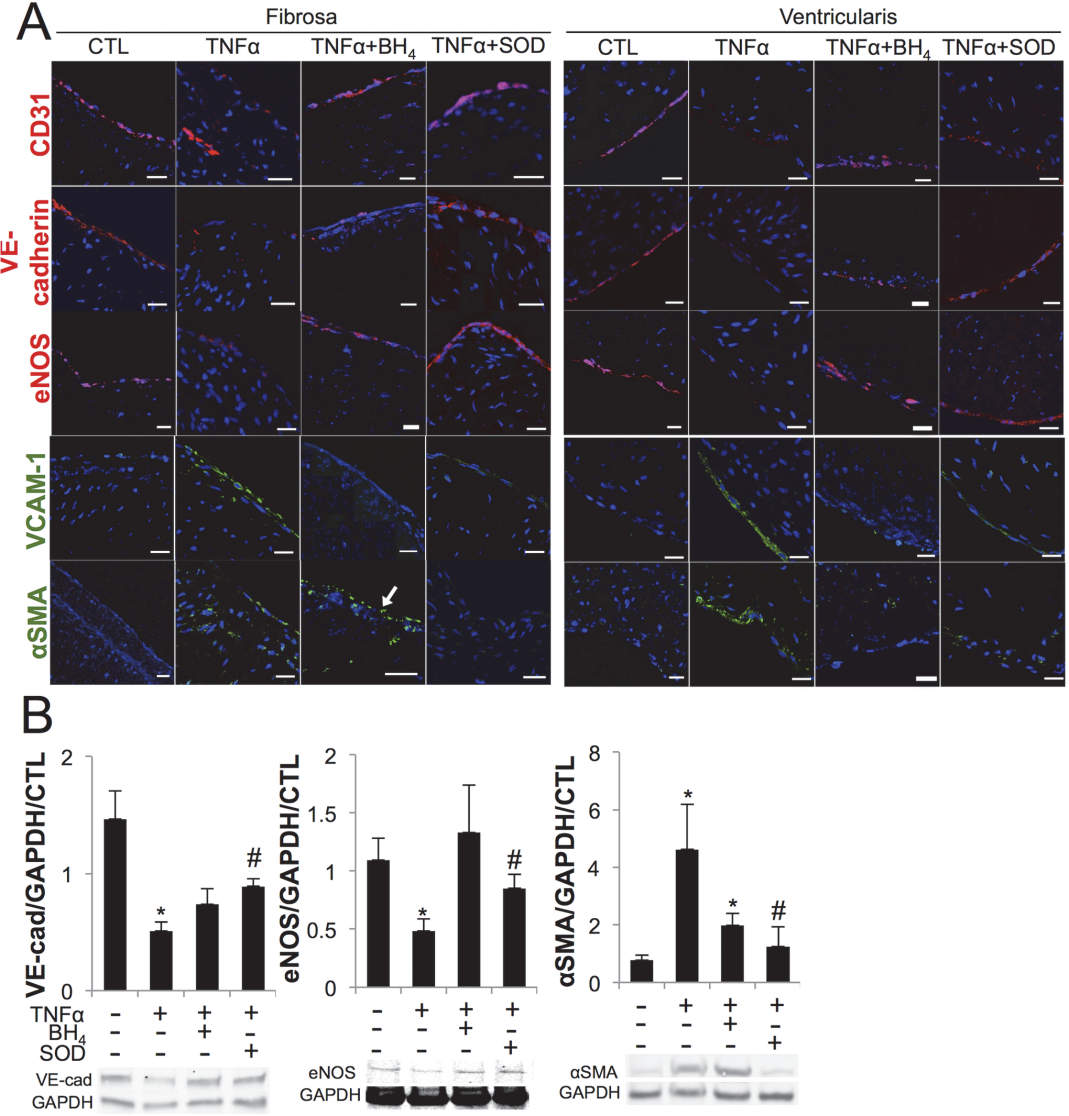
TNFα induces endothelial dysfunction, increased inflammatory activation, and myofibroblastic activation *ex vivo*, rescued by antioxidants. **A**, In *ex vivo* porcine aortic valve leaflets, TNFα decreased endothelial proteins CD31, VE-cadherin, and eNOS on both sides of the valve, rescued by BH_4_ or peg-SOD. TNFα increased VCAM1 on both sides of the valve, mitigated by BH_4_ or peg-SOD. TNFα increased αSMA on both sides of the valve, which was mitigated on the fibrosa by peg-SOD but not by BH_4_ (arrow) and on the ventricularis by either BH_4_ or SOD. **B**, Western blot quantification confirmed loss of VE-cadherin and eNOS and increase in αSMA in TNFα condition that is rescued by BH_4_ or peg-SOD. * indicates p < 0.05 versus CTL, # indicates p < 0.05 versus TNFα. Scale bar = 20μm. N = 6.

AV leaflets cultured in TNFα for 21 days showed significant endothelial dysfunction, with decreases in CD31, VE-cadherin, and eNOS on both the fibrosa and the ventricularis. These effects were rescued by co-treatment with BH_4_ or peg-SOD. TNFα also caused upregulation of VCAM-1 on both sides of the valve, which was mitigated almost entirely on both the fibrosa and ventricularis by co-treatment with BH_4_ or peg-SOD. TNFα significantly increased αSMA in both the endothelium and the underlying interstitium on both sides of the valve, decreased on the fibrosa by peg-SOD and on the ventricularis by BH_4_ or peg-SOD (Fig 6A). Loss of VE-cadherin and eNOS and increases in αSMA due to TNFα and the protective effect of antioxidants were confirmed via western blot analysis (Fig 6B and S6 Fig).

### TNFα causes extracellular matrix disorganization and calcification in aortic valve leaflets, rescued by antioxidants

**Fig 7:**
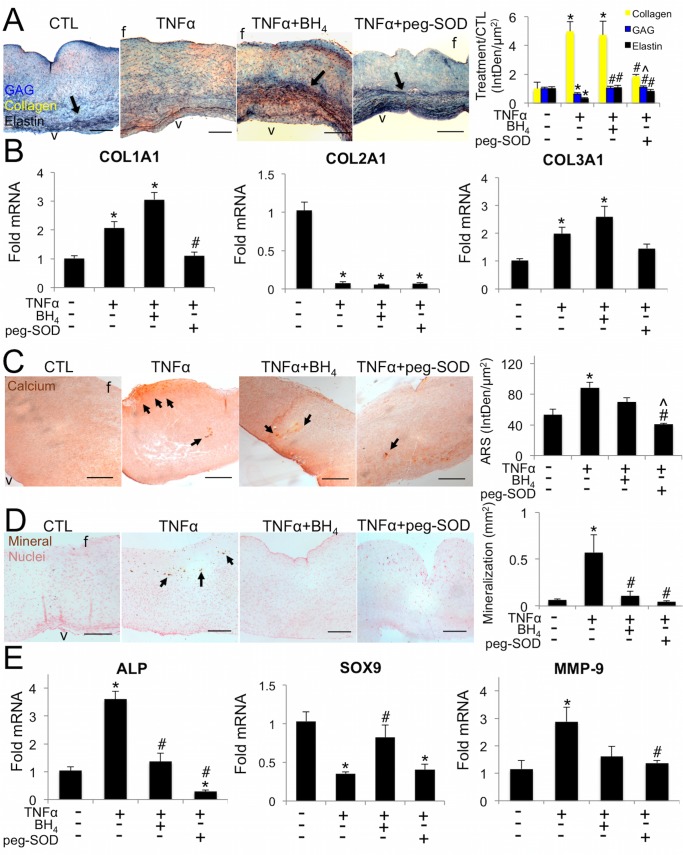
TNFα induces early calcification and changes in aortic valve extracellular matrix composition and structure, rescued differentially by BH_4_ or peg-SOD. **A,** TNFα increased collagen and decreased GAG and elastin (arrows). BH_4_ maintains GAG and elastin at control levels, but does not block increases in collagen. Peg-SOD significantly reduced collagen compared to TNFα and maintained control levels of GAG and elastin (Blue: glycosaminoglycans, Red: mucins, Yellow: collagen, Black, elastin). **B**. TNFα increased synthesis of COL1A1 and COL3A1 mRNA, rescued by peg-SOD but not by BH_4_. COL2A1 was decreased in all treatment conditions. * indicates p < 0.05 versus control, # indicates p < 0.05 versus TNFα. N = 6. One-way ANOVA with Tukey’s post hoc test were used to compare means. **C,** TNFα increased calcium deposition (Alizarin Red S stain) that is rescued by BH_4_ or peg-SOD. **D**, TNFα increased mineralization (von Kossa stain), rescued by BH_4_ or peg-SOD. **E**, TNFα increased alkaline phosphatase, decreased Sox9, and increased MMP-9 mRNA, rescued differentially by BH_4_ or peg-SOD. Representative images from n = 6 leaflets per condition. Scale bar = 200μm. * indicates p < 0.05 versus control, # indicates p < 0.05 versus TNFα, ^ indicates p < 0.05 versus TNFα+BH_4_. N = 6.

*Ex vivo* aortic valve leaflets treated with TNFα for 21 days showed significant fibrogenesis and osteogenesis compared to controls. Control AV leaflets showed distinct trilaminar structure of the extracellular matrix (ECM), with well-defined organization of elastin fibers within the ventricularis (arrow, Fig 7A). Valve leaflets treated with TNFα showed ECM disorganization, marked by significantly decreased elastin and glycosaminoglycans (GAG), and significantly increased collagen. q-rtPCR analysis of the valve tissue showed increased synthesis of COL1A1 and COL3A1 mRNA with TNFα (Fig 7B). BH_4_ protected against loss of elastin and GAG, but allowed similar increases in collagen as in TNFα alone. Peg-SOD protected against loss of elastin and GAG and provided significant protection against increases in collagen, maintaining collagen expression at control levels. Peg-SOD blocked increases in COL1A1 and COL3A1 mRNA. COL2A1 was decreased in all treatment conditions.

Alizarin red staining showed significant increase in free calcium in leaflets cultured with TNFα, localized to the fibrosa side of the leaflets (Fig 7C, arrows) that was mitigated by BH_4_ and returned to control levels by peg-SOD. von Kossa staining showed an increase in mineralization, again on the fibrosa, in leaflets cultured with TNFα that was equally blocked by BH_4_ or peg-SOD (Fig 7D). BH_4_ caused minimal off-target effects on a range of markers regulating AV function (S7 Fig).

TNFα increased synthesis of mRNA for alkaline phosphatase, an enzyme involved in active bone formation, which was mitigated by co-treatment with BH_4_ or peg-SOD. TNFα decreased synthesis of mRNA for the anti-osteogenic transcription factor Sox9, indicating a pro-calcific phenotypic shift that was rescued by co-treatment with BH_4_. TNFα also increased synthesis of mRNA for matrix metalloproteinase-9, mitigated by BH_4_ or peg-SOD (Fig 7E).

## Discussion

**Fig 8:**
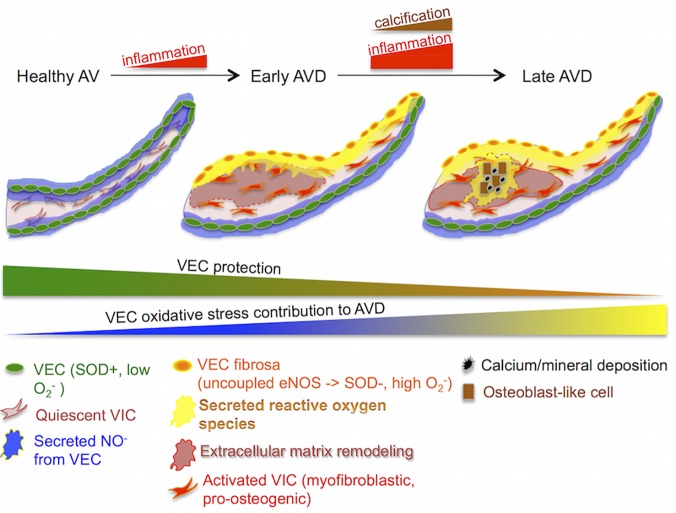
Valve endothelial oxidative stress contributes directly to aortic valve disease. In a healthy valve, endothelial cells secrete protective NO, maintaining quiescence of valve interstitial cells. Early AVD is characterized by inflammation-driven side specific increase in VEC oxidative stress, in part via eNOS uncoupling, causing disorganization of the extracellular matrix and loss of protective NO secretion in the fibrosa. In late AVD, oxidative stress from VEC contributes to calcification and mineral deposition, while NO and SOD are decreased in fibrosa VEC.

Aortic valve disease is a pressing medical issue with few clinically useful pharmacological intervention strategies. New therapies are needed that target specific cellular mechanisms that will guard against valve degeneration. In this study, we have shown the valve endothelium to be a novel source of oxidative stress in the aortic valve when stimulated with the inflammatory cytokine TNFα (Fig 8). We further showed that targeting this mechanism via antioxidants directed at superoxide levels protects against loss of endothelial function, chronic inflammation response, myofibroblastic activation, calcification, and extracellular matrix disorganization.

We showed that regions of the calcified valve endothelium with high VCAM-1 co-localized with regions of high superoxide, linking inflammation with oxidative stress in the diseased valve endothelium. This phenomenon occurs in both mildly and heavily affected regions of the valve, specifically on the fibrosa side of the leaflet. This pervasive and side-specific co-localization of inflammatory activation and oxidative stress in the endothelium suggests that TNFα-driven endothelial oxidative stress occurs throughout mild and advanced stages of AVD. Side-specificity of endothelial oxidative stress points to a distinct involvement of this endothelial-specific mechanism with calcific lesion formation, known to develop preferentially on the fibrosa [36]. Our observation of increased superoxide levels in hVEC but not the adjacent hVIC supports a role for valve endothelial cells as a unique source of oxidative stress in the diseased valve, distinct from oxidative stress previously described in the calcifying regions of the valve [23–24] or as a result of immune cell invasion. We also observed fibrosa-specific loss of SOD1 expression in cHAV, indicating a dearth of SOD1 in the diseased fibrosa endothelium during CAVD, making the valve more susceptible to increases in oxidative stress from the endothelium. Low fibrosa SOD1 compared to the ventricularis in cHAV can also explain the preferential accumulation of superoxide that we observed in the fibrosa endothelium of calcified valves.

Recently, TNFα signaling has been implicated as a key inflammatory pathway in stenotic aortic valves [37–39]. Mouse models of hypercholesterolemia show inflammatory activation of the endothelium, increased superoxide [40], and endothelial dysfunction [41]. Here, we have demonstrated that TNFα increases endothelial oxidative stress, decreases NO secretion, and lowers VE-cadherin and eNOS protein expression in valve endothelial cells, both *in vitro* and *ex vivo*. This finding provides a mechanistic link between inflammation, endothelial dysfunction, and oxidative stress. TNFα-derived oxidative stress effects were blocked by co-treatment with eNOS co-factor BH_4_, demonstrating that eNOS uncoupling is a primary mechanism of increased endothelial oxidative stress and a significant cause of endothelial dysfunction that leads to increased AV calcification via degradation of endothelial protection.

Co-treatment of VEC with TNFα+catalase did not protect the endothelium, but led to increased cellular apoptosis and elevated inflammatory activation. This agrees with previous studies that have shown that in certain cell types, H_2_O_2_ actually protects against TNFα –induced apoptosis and that increased levels of catalase has a neutral or negative effect [42–44]. The ability of apocynin to mitigate TNFα-driven endothelial oxidative stress levels as indicated by DCF staining shows that NADPH oxidases also have a role to play in this phenomenon, though it appears to be less important than the uncoupling mechanism, as demonstrated by the more potent effects of BH_4_. These findings define two unique mechanisms of endothelial oxidative stress contribution to AVD: via changes in eNOS activity due to uncoupling and disruption of native SOD1 and H_2_O_2_ levels. TNFα driven oxidative stress, in part due to eNOS uncoupling, degrades the endothelial protection that would normally mitigate valve degeneration [15, 17] and increases oxidative stress in the valve. This establishes a novel mechanism of valve oxidative stress based on endothelial inflammatory activation, distinct from existing descriptions of oxidative stress that have focused on valve interstitial cells [25, 45–47].

We further established the ability of BH_4_ and peg-SOD to mitigate increases in VCAM-1 that occurred in VEC in response to TNFα. This dovetails with recent studies that implicate side-specific VCAM-1 expression in the progression and regulation of AVD [48–49]. Thus, mitigating the cellular stress imposed on VEC by TNFα-driven eNOS uncoupling may also decrease the need to activate downstream inflammatory signaling pathways. Mitigation of the symptoms of chronic inflammation could prove important to aortic valve calcification, as the TNFα signaling pathway is known to promote osteogenesis [50] and to be an active regulator of lesion formation in the vasculature [51].

Translation of our cellular-level findings into an *ex vivo* evaluation of AV leaflets allowed us to assess the specific effects of TNFα and antioxidants on the complex, multicellular aortic valve leaflet. We found that superoxide levels in TNFα-treated valve leaflets was specifically elevated in VEC, with highest elevation on the fibrosa side, confirming that endothelial superoxide in cHAV is linked to inflammation of the valve endothelium. Interestingly, co-treatment of *ex vivo* AV leaflets with TNFα+BH_4_ reduced oxidative stress only in the fibrosa endothelium compared to TNFα alone, whereas peg-SOD reduced oxidative stress on both sides of the valve leaflet. Examination of mitochondrial ROS provided an explanation for this difference, as peg-SOD but not BH_4_ lowered mtROS levels within the ventricularis of *ex vivo* leaflets. We conclude that BH_4_ treatment mitigates valve oxidative stress via direct targeting of fibrosa-specific eNOS uncoupling, whereas peg-SOD mitigates both fibrosa-specific eNOS uncoupling and mtROS within ventricularis VEC. A limitation of this study is the lack of direct experimental interrogation of mtROS as a propagator of AVD; however, the evidence presented here provides a foundation for future investigation of mitochondrial contribution to increased oxidative stress in the aortic valve in the early inflammatory stages of AVD.

Immunofluorescent staining confirmed the side-specific effect of BH_4_. CD31, VE-cadherin, and eNOS were more robustly restored on the fibrosa with co-treatment of TNFα+BH_4_, compared to TNFα alone. TNFα caused increased αSMA in the fibrosa, potentially due to mesenchymal transition of valve endothelial cells [34], in addition to direct myofibroblastic activation of valvular interstitial cells [31]. TNFα-induced increases in αSMA were blocked by co-treatment of valve leaflets with TNFα+BH_4_ or TNFα+peg-SOD, demonstrating a role for both eNOS uncoupling and superoxide from other sources such as NADPH oxidases and mtROS in driving myofibroblastic activation during AVD.

TNFα exposure caused significant disruption of the extracellular matrix organization and content of the aortic valve. TNFα increased collagen and decreased GAG and elastin, agreeing with studies that find differential GAG localization [52] and reduced elastin [53] in diseased aortic valves. GAG and elastin were preserved by BH_4_ or peg-SOD. Increases in collagen were only mitigated by peg-SOD, not by BH_4_. mRNA analysis showed that increased collagen was caused by increased expression of COL1A1 and COL3A1 isoforms in TNFα and TNFα+BH_4_ conditions, but rescued in TNFα+peg-SOD condition. Interestingly, COL2A1 was uniformly decreased across TNFα, TNFα+BH_4_, and TNFα+peg-SOD conditions, pointing to TNFα as a driver of early chondrocyte differentiation in valve cells and as an important contributor to valve calcification via a mechanism separate from the oxidative stress pathways examined here [54]. These findings indicate that protection against excess valve endothelial oxidative stress can guard against ECM changes known to feature in AVD [55] via antioxidant mitigation of inflammation, endothelial dysfunction, and oxidative stress.

TNFα accelerates calcification of aortic valve interstitial cells *in vitro* [32–33]. However, the contribution of TNFα to AVD within the complex multi-cellular environment of the native aortic valve is unknown. Here we used TNFα with and without BH_4_ treatment to show that stimulation of endothelial eNOS uncoupling by TNFα contributes to valve calcification, elucidating a novel mechanism of TNFα contribution to AVD. Increases in alkaline phosphatase point to the mechanism behind these changes, indicating that TNFα stimulates early osteogenic differentiation of valve interstitial cells, as has been observed *in vitro* under TGF-β stimulation [56], that can be protected against by preservation of endothelial function and reductions in oxidative stress.

Antioxidant therapy for AVD must be founded on specific mechanisms generating oxidative stress in order to be efficacious [57]. The evidence presented here establishes inflammatory activation of endothelial oxidative stress as a unique integrated pathobiological mechanism effecting early to late stages of aortic valve disease. Our findings support that specific targeting of eNOS uncoupling and the resulting excess superoxide may be key elements of managing AVD via antioxidant treatments.

## Supporting Information

S1 Fig TNFα caused increased superoxide expression in VEC. A, Intracellular superoxide in VEC+TNFα at 30 minutes after treatment. Presented using colorimetric scale to show relative DHE intensity, indicated in inset triangle. B, Quantification of superoxide production at 30 minutes using microplate assay for DHE fluorescence. * indicates p < 0.05 versus control. N > 6 for each condition. DHE fluorescence was measured using integrated pixel density in three different fields of view (250μm^2^) on each sample. The three measurements were averaged within each sample, with each average corresponding to N of 1. Means were compared using unpaired Student’s t-test, assuming unequal variance.
(TIFF)

S2 Fig Original western blot images for Fig 3. PAVEC cultured 48 hrs on 3D hydrogels with control, +30 ng/mL TNFα, or 1μM H_2_O_2_. Boxed regions indicate bands shown in Fig 3.
(TIFF)

S3 Fig VEC cultured *in vitro* on 3D hydrogels for 48 hours with TNFα+antioxidants. A. TNFα+catalase treatment caused a significant increase in apoptosis. B. TNFα+catalase treatment caused increased loss of VE-cadherin, increased nuclear translocation of NFkB, and increased VCAM-1 expression compared to both control and TNFα alone. N = 3, * indicates p < 0.05 vs CTL, # indicates p < 0.05 vs TNFα. Scale bar is 20μm.
(TIFF)

S4 Fig Original western blot images for Fig 4. PAVEC cultured 48 hrs on 3D hydrogels with control, +30 ng/mL TNFα, +30 ng/mL TNFα+10μM BH_4_, or +30 ng/mL TNFα+20U/mL peg-SOD. Boxes indicate bands shown in Fig 4. *Analysis of NFκB p65 protein expression was not used in this study.
(TIFF)

S5 Fig Apoptosis in *ex vivo* AV leaflets cultured for 21 days. There was no significant apoptosis in any of AV leaflets, as measured by the TUNEL assay. N = 6 for all sample groups. Means were compared using one-way ANOVA with Tukey’s post hoc test.
(TIFF)

S6 Fig Original western blot images for Fig 6. Porcine AV leaflets cultured 21 days in control, +30 ng/mL TNFα, +30 ng/mL TNFα+10μM BH_4_, or +30 ng/mL TNFα+20U/mL peg-SOD. Boxes indicate bands shown in Fig 6. *Analysis of NFκB p65 protein expression was not used in this study.
(TIFF)

S7 Fig q-rtPCR analysis of *ex vivo* AV leaflets with TNFα+antioxidants. A. Complex regulation of transcription factors Runx2 and Msx2 by TNFα and BH_4_ or SOD. N = 6, * indicates p < 0.05 vs CTL, # indicates p < 0.05 vs TNFα. B. BH_4_ co-treatment consistently causes either neutral or beneficial effects compared to TNFα alone. N = 6, * indicates p < 0.05 vs TNFα.
(TIFF)

S1 Supplement Supplemental methods. (DOCX)
