# Correction: Endothelial-Derived Oxidative Stress Drives Myofibroblastic Activation and Calcification of the Aortic Valve

**DOI:** 10.1371/journal.pone.0128850

**Published:** 2015-05-18

**Authors:** Emily J. Farrar, Geoffrey D. Huntley, Jonathan Butcher

Fig 3 is incorrect in panels E through I. The authors have provided a corrected version here.

**Fig 3:**
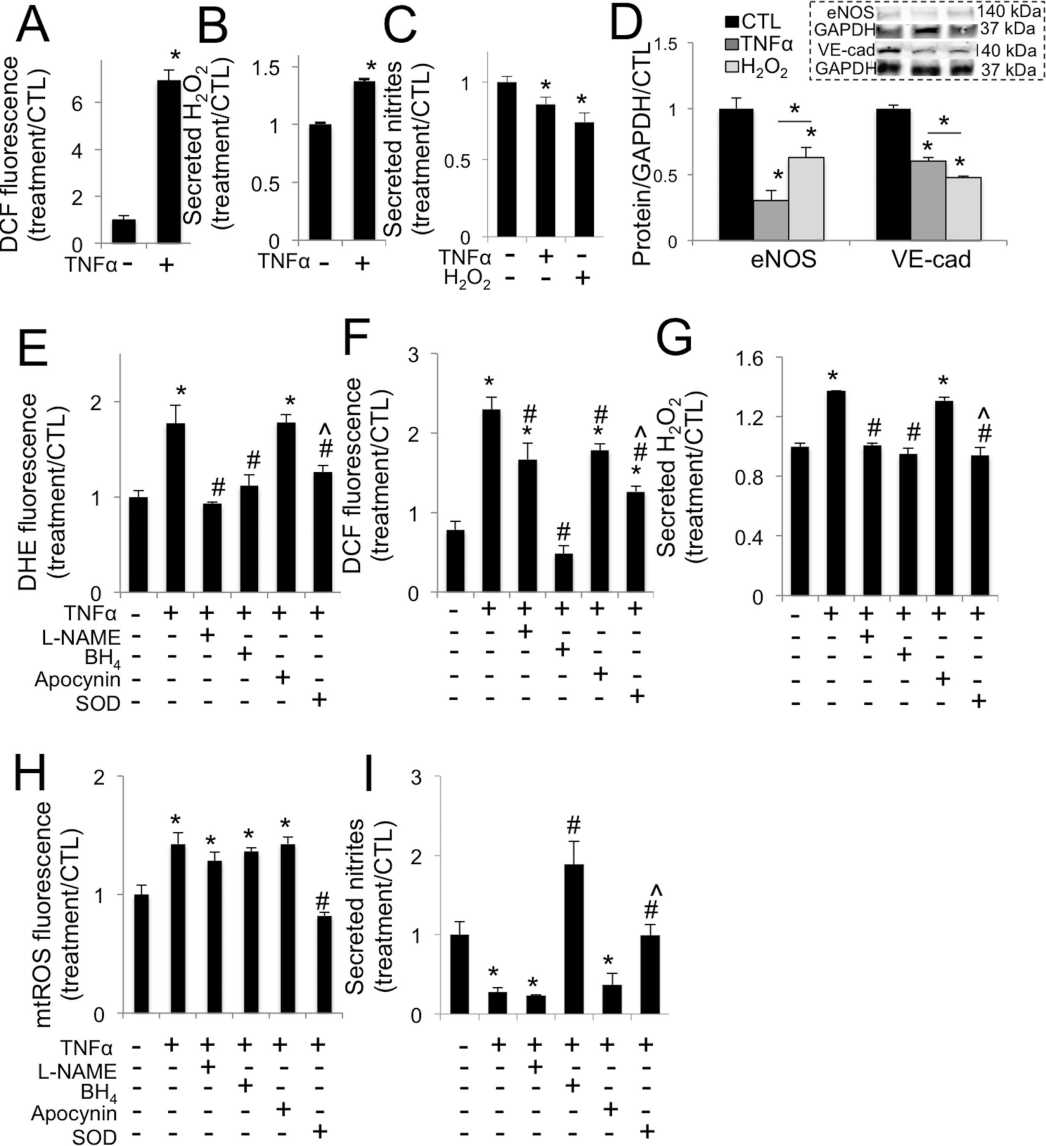
TNFα drives increased oxidative stress in aortic valve endothelial cells via eNOS uncoupling. **A**, TNFα increases oxidative stress in VEC at 30 minutes. **B**, TNFα increases hydrogen peroxide (H_2_O_2_) secretion from VEC at 30 minutes. **C**, TNFα or H_2_O_2_ decrease nitric oxide secretion from VEC at 48 hours (n = 4). **D**, TNFα or H_2_O_2_ decrease eNOS and VE-cadherin expression in VEC at 48 hours. Representative western blot images (inset) and blot quantification. **E**, L-NAME, BH_4_, or peg-SOD but not apocynin block increases in superoxide (DHE) in VEC caused by TNFα, at 30 minutes. **F**, L-NAME, apocynin, and peg-SOD mitigate increases in general oxidative stress (DCF) caused by TNFα at 30 minutes, but only BH_4_ completely blocks superoxide increase, maintaining control levels. **G**, L-NAME, BH_4_, or peg-SOD but not apocynin block increases in H_2_O_2_ secreted by VEC at 30 minutes caused by TNFα at 30 minutes. **H**, TNFα drives increased mtROS, mitigated only by co-treatment with SOD. **I**, BH_4_, or peg-SOD but not L-NAME or apocynin block decreases in nitric oxide secretion in VEC caused by TNFα at 48 hours. * indicates p < 0.05 versus control. # indicates p < 0.05 versus TNFα. ^ indicates p < 0.05 versus apocynin. N = 4.
