# Supplementary figures and images for: Endothelial-Derived Oxidative Stress Drives Myofibroblastic Activation and Calcification of the Aortic Valve
Source: PLoS One. 2015 Apr 13;10(4):e0123257. doi: 10.1371/journal.pone.0123257 (PMC4395382; doi:10.1371/journal.pone.0123257)

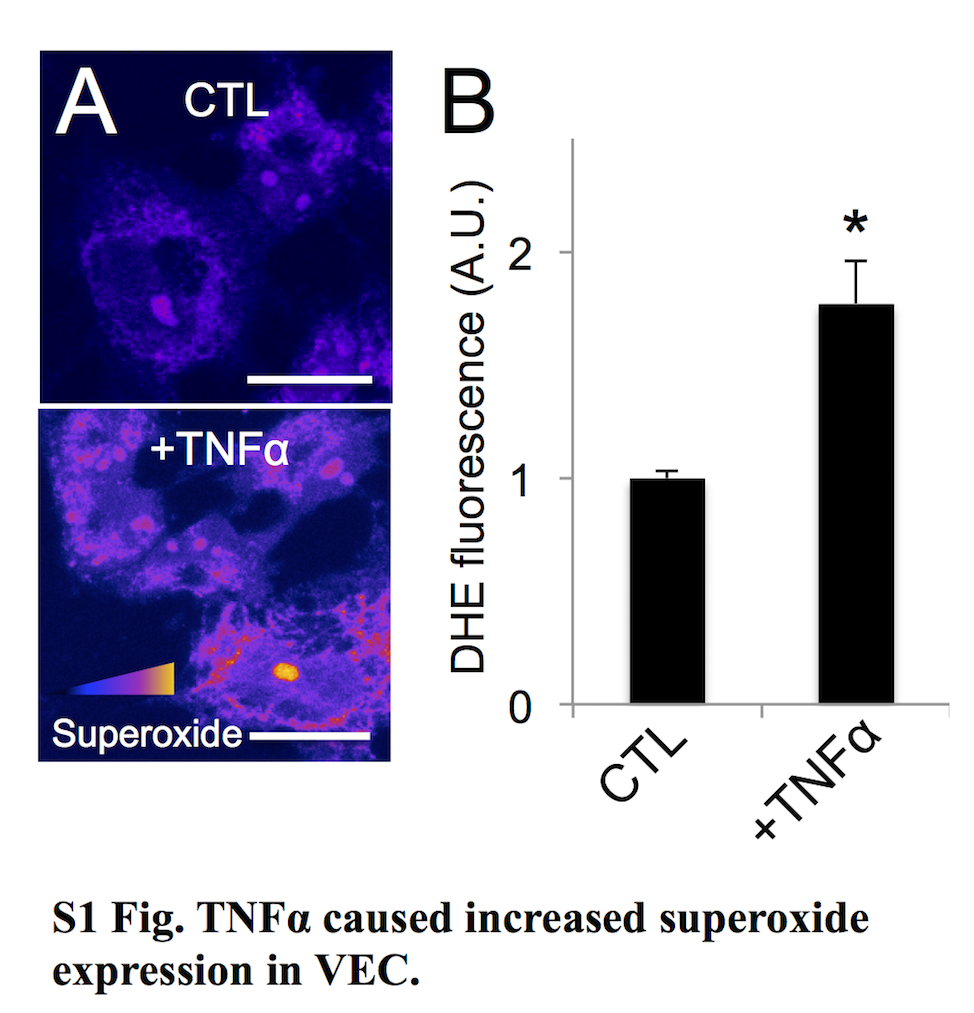

Supplement: S1 Fig — A, Intracellular superoxide in VEC+TNFα at 30 minutes after treatment. Presented using colorimetric scale to show relative DHE intensity, indicated in inset triangle. B, Quantification of superoxide production at 30 minutes using microplate assay for DHE fluorescence. * indicates p < 0.05 versus control. N > 6 for each condition. DHE fluorescence was measured using integrated pixel density in three different fields of view (250μm2) on each sample. The three measurements were averaged within each sample, with each average corresponding to N of 1. Means were compared using unpaired Student’s t-test, assuming unequal variance. (TIFF) [file pone.0123257.s001.tiff]

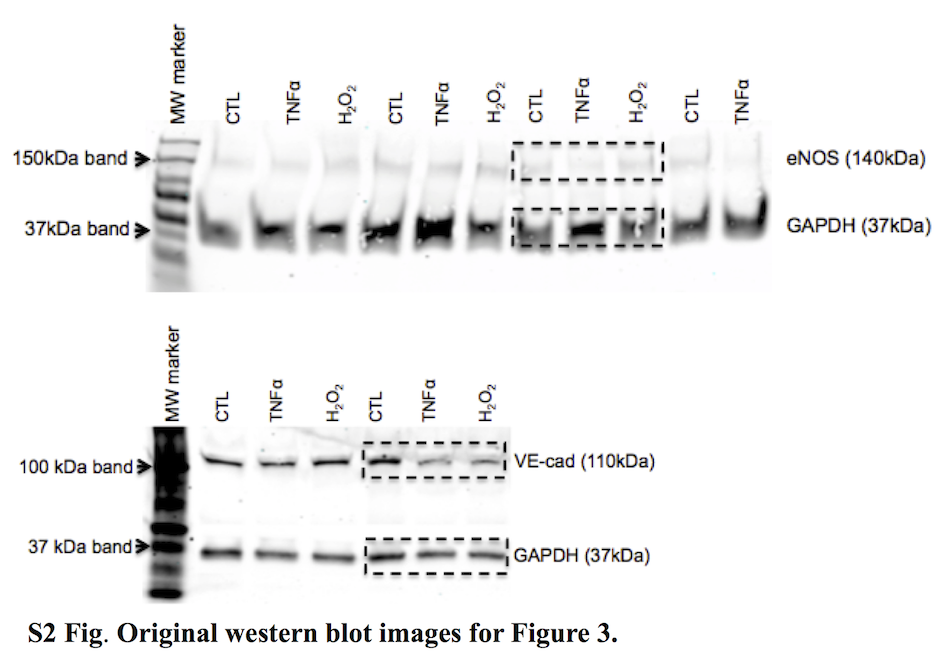

Supplement: S2 Fig — PAVEC cultured 48 hrs on 3D hydrogels with control, +30 ng/mL TNFα, or 1μM H2O2. Boxed regions indicate bands shown in Fig 3. (TIFF) [file pone.0123257.s002.tiff]

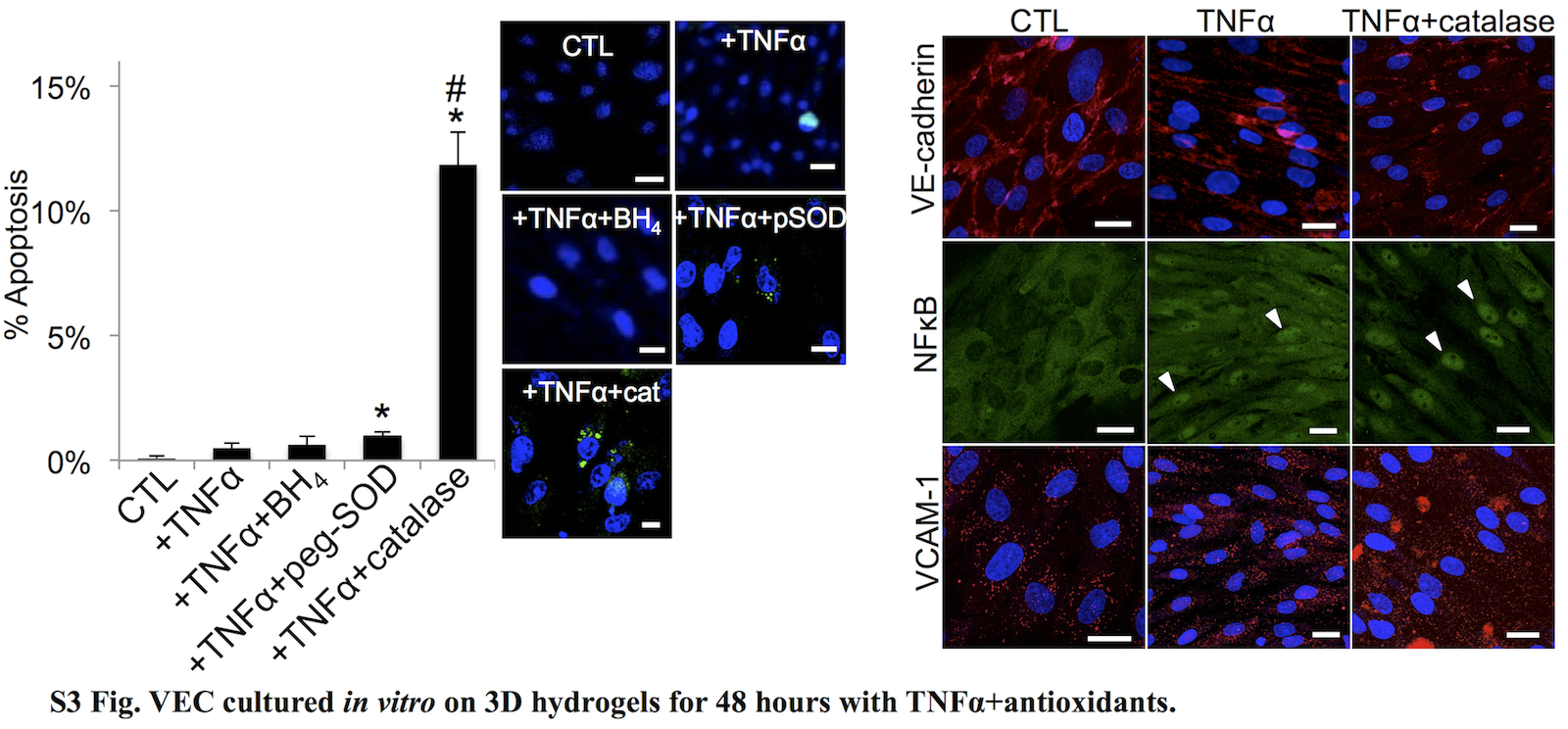

Supplement: S3 Fig — A. TNFα+catalase treatment caused a significant increase in apoptosis. B. TNFα+catalase treatment caused increased loss of VE-cadherin, increased nuclear translocation of NFkB, and increased VCAM-1 expression compared to both control and TNFα alone. N = 3, * indicates p < 0.05 vs CTL, # indicates p < 0.05 vs TNFα. Scale bar is 20μm. (TIFF) [file pone.0123257.s003.tiff]

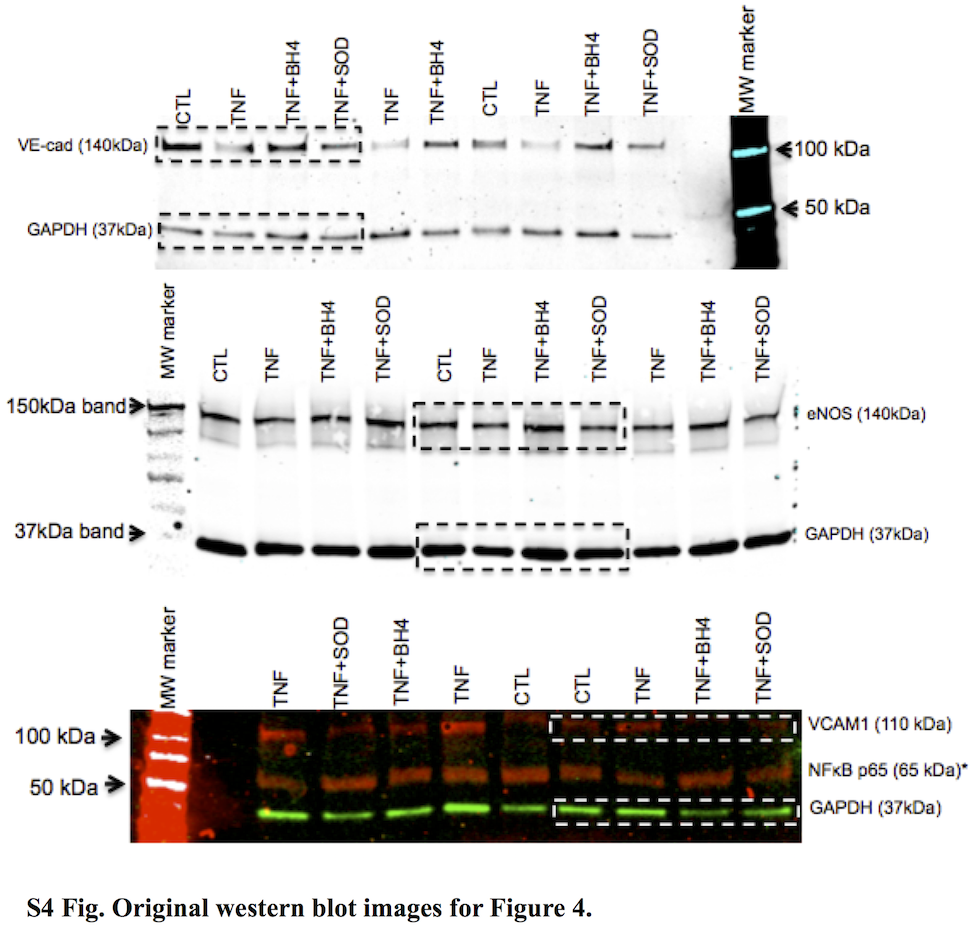

Supplement: S4 Fig — PAVEC cultured 48 hrs on 3D hydrogels with control, +30 ng/mL TNFα, +30 ng/mL TNFα+10μM BH4, or +30 ng/mL TNFα+20U/mL peg-SOD. Boxes indicate bands shown in Fig 4. *Analysis of NFκB p65 protein expression was not used in this study. (TIFF) [file pone.0123257.s004.tiff]

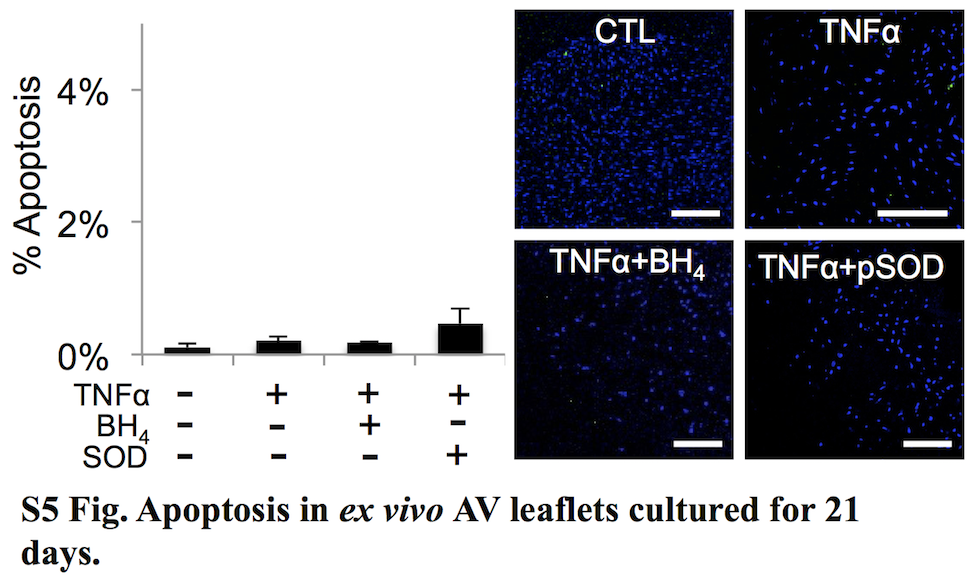

Supplement: S5 Fig — There was no significant apoptosis in any of AV leaflets, as measured by the TUNEL assay. N = 6 for all sample groups. Means were compared using one-way ANOVA with Tukey’s post hoc test. (TIFF) [file pone.0123257.s005.tiff]

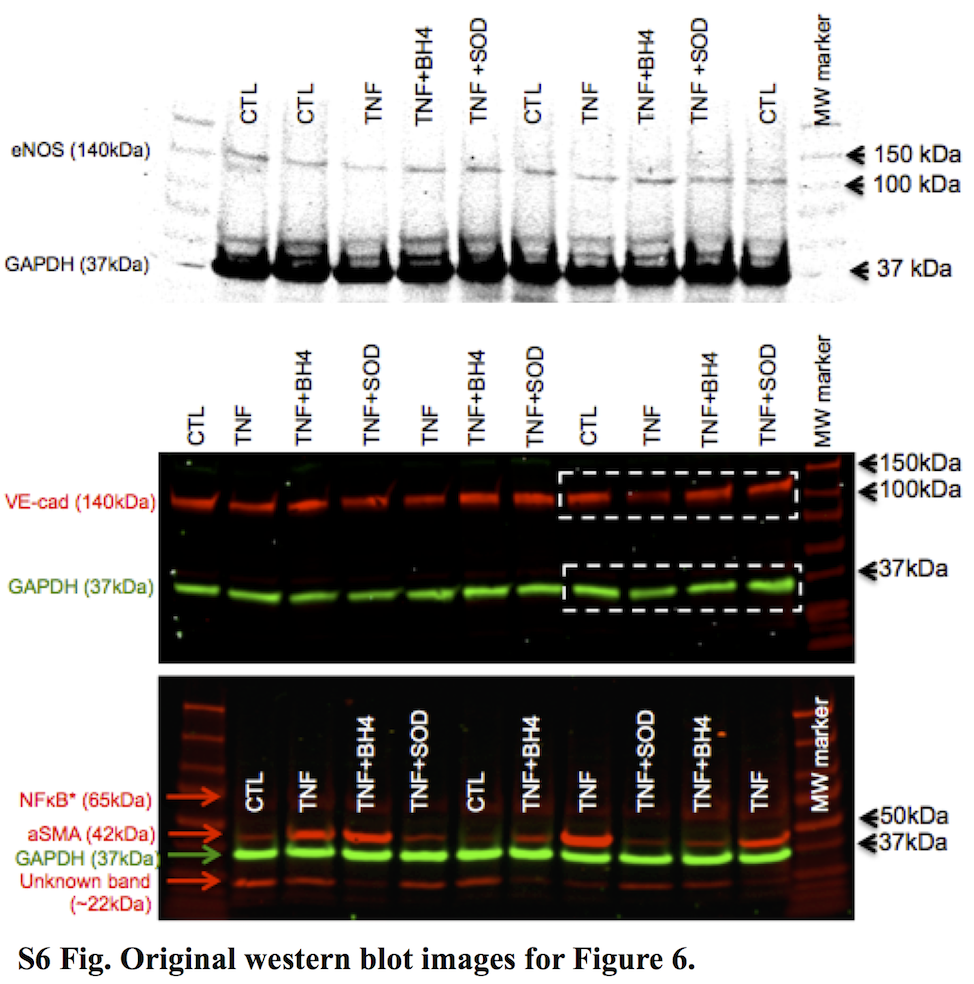

Supplement: S6 Fig — Porcine AV leaflets cultured 21 days in control, +30 ng/mL TNFα, +30 ng/mL TNFα+10μM BH4, or +30 ng/mL TNFα+20U/mL peg-SOD. Boxes indicate bands shown in Fig 6. *Analysis of NFκB p65 protein expression was not used in this study. (TIFF) [file pone.0123257.s006.tiff]

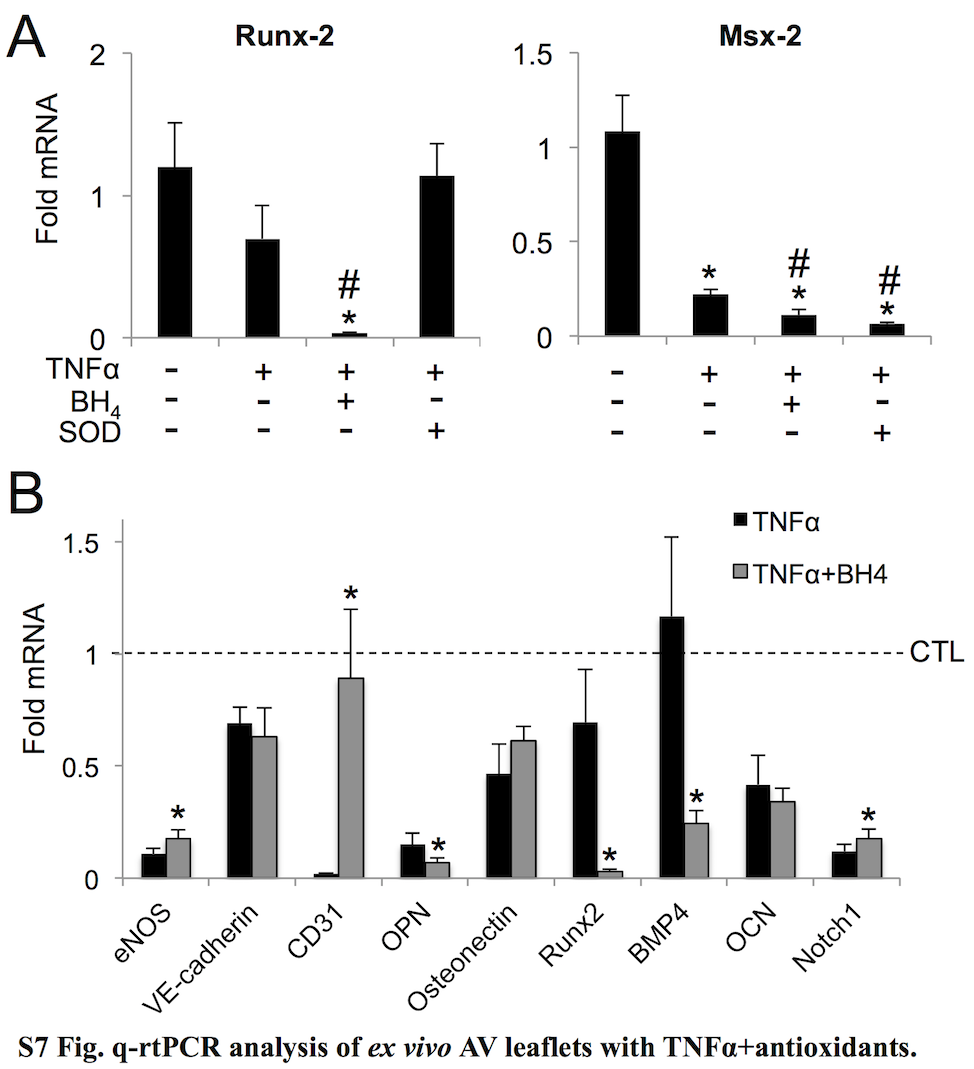

Supplement: S7 Fig — A. Complex regulation of transcription factors Runx2 and Msx2 by TNFα and BH4 or SOD. N = 6, * indicates p < 0.05 vs CTL, # indicates p < 0.05 vs TNFα. B. BH4 co-treatment consistently causes either neutral or beneficial effects compared to TNFα alone. N = 6, * indicates p < 0.05 vs TNFα. (TIFF) [file pone.0123257.s007.tiff]
