# Supplementary material for: Endothelial-Derived Oxidative Stress Drives Myofibroblastic Activation and Calcification of the Aortic Valve
Source: PLoS One. 2015 Apr 13;10(4):e0123257. doi: 10.1371/journal.pone.0123257 (PMC4395382; doi:10.1371/journal.pone.0123257)
Supplement: S1 Supplement — (DOCX) [file pone.0123257.s008.docx]

**Supplemental Methods**

**Human aortic valve samples**

Human diseased aortic valves were obtained from adults undergoing planned, non-elective valve replacement surgery at Robert Packer Hospital in Sayre, PA. Of all valves used, patient age range was 70-90 years, with a mean age of 77 years. 33% were female; all were diagnosed with moderate to severe aortic stenosis; one patient had concurrent peripheral vascular disease. There were no racial/ethnic-based differences in samples. Healthy, non-diseased aortic valves were obtained from patients who died from non-valve related illnesses, who had no visible valve pathology, and whose hearts were ineligible for organ donation due to non-valve related defects, provided by Dr. Jonathan Chen, Cornell-Weill Medical School and NY Presbyterian Hospital. Institutional Review Boards at Cornell University, NY Presbyterian Hospital and Robert Packer Hospital approved all procedures. All samples were procured with informed consent from patients.

For quantification of superoxide, VCAM-1, and superoxide dismutase in the valve endothelium, CD31 expression was used to detect presence or absence of endothelial cells along each point on the valve periphery. Superoxide, VCAM-1, and superoxide dismutase expression were quantified at each point on the periphery that was positive for CD31. This value was integrated over the fibrosa or the ventricularis endothelium and calculated as integrated density of SOD or VCAM-1 expression per micron.

**Superoxide in human aortic valve sections**

Calcified human aortic valve (cHAV) leaflets were decalcified in 10% sodium citrate and 22% formic acid in deionized water for 12 hours or until soft, then washed in running water for 30 minutes. Decalcified cHAV and non-diseased HAV were embedded in paraffin wax and sectioned in 6μm increments. Superoxide was visualized using dihydroethidium (DHE) fluorescence. Valve sections were deparaffinized, washed in 1x Hank’s buffered salt solution (HBSS) (Invitrogen), and incubated in the dark in 10 μmol/L DHE for 30 minutes. Sections were rinsed twice and imaged immediately on a Zeiss 710 (Thornwood, NY) laser scanning confocal microscope, using 488nm excitation and 585nm emission. Images were adjusted to minimize background fluorescence using controls and fluorescence intensity was mapped onto a colorimetric intensity scale using ImageJ (NIH).

**In vitro aortic valve endothelial cell isolation and culture**

Porcine valves are widely used as an analog for healthy human adult valve endothelial cells [1]. Unlike mice, pigs develop atherosclerosis and valve lesions without intervention, similar to humans [2]. Large-scale culture of porcine valve endothelial cells is feasible due to the ample size of the valve; to date a pure population of mouse valve endothelial cells has never been demonstrated. Endothelial cells from porcine valves used here were screened for purity of phenotype, assuring a consistent match with known characteristics of healthy human valves, including no α−SMA expression and cobblestone morphology. This is advantageous in light of recent studies that have shown elevated α−SMA expression in valve cells obtained from adult human transplants, suggesting variability in phenotype [3].

All reagents obtained from Sigma-Aldrich, St Louis, MO, unless otherwise noted. Porcine aortic valve endothelial cells (PAVEC) were isolated as demonstrated previously^16^ from valves donated by Shirk Meats of Dundee, NY. PAVEC were grown in flasks coated with 50 μg/mL rat-tail collagen I (BD Biosciences, San Jose, CA) at 37^o^C and 5% CO2 in DMEM supplemented with 10% FBS (Invitrogen, Grand Island, NY), 1% penicillin-streptomycin (Invitrogen, Grand Island, NY), and 50U/mL heparin. Cells were passaged 1:3 at confluency using 0.25% Trypsin-EDTA (Invitrogen, Grand Island, NY) and media was changed every 48 hours. Purity of endothelial population was monitored via quantitative real-time PCR, western blot, and immunofluorescent assessment. Only cultures with consistent CD31 and VE-cadherin expression, cobblestone morphology, and non-detectable αSMA expression were used. αSMA levels were measured via real-time PCR (> 37 cycle threshold), western blot, and immunofluorescence. PAVEC cultures were used between passage four and six. For all experiments and assays except those requiring protein isolation for Western blot, PAVEC were cultured on 3-D collagen hydrogels composed of 10% FBS (Gemini Biosciences, West Sacramento, CA), 1x Dulbecco’s modified eagle medium (D-MEM, Invitrogen, Grand Island, NY), and 2 mg/mL collagen type I (BD Biosciences, San Jose, CA), adjusted to a pH of 7.2. After one hour incubation of the hydrogels at 37°C, PAVEC were seeded at 100,000 cells/cm^2^ and allowed to adhere overnight. For Western blot, PAVEC were cultured in 6-well plates to 70% confluence, and then treatment was added. For treatments, cells were rinsed twice in fresh 1x PBS, then human recombinant TNF-α or 1μmol/L H2O2 in DMEM was added. 500μmol/L L-NAME was added 30 minutes prior to TNF-α treatment; all other co-treatments were added concurrently with TNF-α, using 1000U/mL catalase, 10 μmol/L BH_4_, 20U/mL pegylated Cu-Zn-Superoxide dismutase, or 100μmol/L Apocynin suspended in DMSO.

**Free radicals**

Nitrite secretion from PAVEC was assessed using the Griess assay (Invitrogen, Grand Island, NY), according to the manufacturer’s instructions. Importantly, heparin was not added to the culture media used for any conditions of the Griess assay, as heparin induces precipitation upon addition of the Griess reagent. Hydrogen peroxide secretion was assessed using the Fluoro H_2_O_2_ kit (Cell Technology, Inc) according to the manufacturer’s instructions. Phenol-red-free D-MEM was used to culture PAVEC and PAEC in all treatments for H_2_O_2_ measurement experiments, in order to optimize signal to noise ratio. Standard curves for each assay were performed using media kept in identical conditions as treatments and controls, but without cells.

CM-H_2_DCFDA (DCF) (Invitrogen, Grand Island, NY), was used to assess general oxidative stress state in PAVEC and PAEC. Cells were cultured overnight on collagen gels, then treatment was added for amount of time specified in text. Samples were then rinsed twice in 1x HBSS and 10μmol/L CM-H_2_DCFDA in 1x HBSS was added and incubated at 37^o^C for one hour. Samples were rinsed twice in 1x HBSS and assessed for dye fluorescence immediately on a Gen5 micro-plate reader (BioTek, Winooski, VT) with 485/525 excitation/emission.

Dihydroethidium (DHE) was used to assess superoxide levels in PAVEC and deparaffinized *ex vivo* aortic valve leaflet sections [4]. Time course imaging studies were performed in order to determine the optimal length of time for DHE stain incubation, as well as the degradation of the signal during imaging. 1 hour of 10uM DHE stain, 2 rinses in HBSS, and immediate imaging was found to give optimum results in terms of minimal background and bright calcific nodule (positive control). Samples were washed twice in 1x Hank’s buffered salt solution (HBSS) (Invitrogen, Grand Island, NY), and incubated in the dark in 10 μmol/L DHE for 30 minutes. Sections were rinsed twice in 1x HBSS and imaged immediately on a Zeiss 710 (Thornwood, NY) laser scanning confocal microscope (488/585) or immediately on a Gen5 (BioTek, Winooski, VT) micro-plate reader (485/525). Confocal images were adjusted to minimize background fluorescence using negative controls and fluorescence intensity was mapped onto a colorimetric intensity scale using ImageJ (NIH). Micro-plate data was adjusted to remove background fluorescence and averaged across at least three samples for each condition.

MitoSOX red mitochondrial superoxide indicator was used to detect mitochondrial O_2_^-^ in both *in vitro* and *ex vivo* samples. Time course imaging studies were performed in order to determine the maximum MitoSOX signal produced from *in vitro* porcine VEC, as well as the optimal length of time for MitoSOX stain incubation, and the degradation of the signal during imaging (negligible over 30 minute imaging period). The *in vitro* protocol for VEC on 3-D hydrogels was as follows: treat VEC gels with TNF-α+antioxidants as described above, incubate for 30 minutes, rinse twice in 1x PBS, add 200μL of 5μM MitoSOX in 1x HBSS to each gel. Incubate 10 minutes at 37^o^C, rinse 3x in 1x HBSS, and image immediately on a Gen5 (BioTek, Winooski, VT) micro-plate reader (510/590). Micro-plate data was adjusted to remove background fluorescence, averaged across at least three samples for each condition, and normalized to control samples.

For *ex vivo* valve leaflets, samples were processed identical to the DHE staining procedure, but with 10 minutes MitoSOX stain incubation at 37^o^C and a final co-stain with 5μM Draq5 (Cell Signaling Technology) for 10 minutes at room temperature. Slides were then rinsed 3x in 1x PBS, mounted with Permount anti-fade mounting media (Life Technologies), and imaged immediately on a Zeiss 710 (Thornwood, NY) laser scanning confocal microscope (488/585). MitoSOX staining intensity was analyzed by separating only the 590nm fluorescence channel and using ImageJ to quantify integrated pixel intensity over different regions of the leaflet: interstitium, fibrosa endothelium, and ventricularis endothelium. Data is presented for n=6 samples per condition and normalized to control leaflet intensities.

**Immunofluorescence**

PAVEC on collagen gels were rinsed twice in PBS and fixed in 4% paraformaldehyde for one hour at 37^o^C or overnight at 4^o^C. Samples were rinsed 3 times on a rocker with PBS for 15 minutes each, permeabilized with 0.2% Triton-X (VWR International, West Chester, PA) for 10 minutes on rocker, and blocked in 10% goat serum in 1% BSA solution for one hour at 37^o^C or overnight at 4^o^C. Samples were rinsed 3x for 15 minutes each in PBS and primary antibodies were added: mouse anti-pig CD31 (AbD Serotec, 1:100), mouse anti-human eNOS (BD transduction laboratories, 1:100), rabbit anti-human VE-cadherin (Cell Signaling Technology, 1:100), rabbit anti-human VCAM-1 (Santa Cruz Biotechnology, 1:50), rabbit anti-human NFκB (Cell Signaling Technology, 1:100), and rabbit anti-human α-SMA (Abcam, 1:100). Samples were incubated overnight at 4^o^C, rinsed in 3x for 15 minutes each in PBS on rocker, and species-specific secondary antibodies raised in goat conjugated to Alexa Fluor® 488 or 568 fluorophores were added. Samples were incubated for 2 hours at room temperature, rinsed, then incubated for an additional 30 minutes with 1:1000 Draq5 nuclear stain (Enzo Life Sciences AG, Lausen, Switzerland). Samples were rinsed thoroughly in PBS 3 times on rocker for 15 minutes each, then imaged using a Zeiss 710 (Thornwood, NY) laser scanning confocal microscope.

**Western blot**

PAVEC were lysed directly on the plate using RIPA buffer (Thermo Scientific, Rockford, IL) supplemented with 25mM NaF, 1mM NaVO_4_, and 0.5% Protease inhibitor cocktail, incubated for 15 min at RT, scraped and homogenized by pipetting up and down, and centrifuged at 15,000 rpm for ten minutes. Protein was mass balanced using Pierce 660nm protein assay with ionic detergent compatibility reagent (Thermo Scientific, Waltham, MA) and loaded into a 4-15% gradient gel (Bio-Rad, Hercules, CA) with 4x buffer (Li-Cor, Lincoln, NE)+10% β-mercaptoethanol at 1:3 ratio and run for one hour at 120V in 25mM Tris, 0.2M Glycine, 1% SDS running buffer. Western blot transfer to nitrocellulose membrane (Thermo Scientific, Rockford, IL) was performed at 400mA for one hour in 25mM Tris, 20% methanol transfer buffer. After rinsing in PBS 0.1% Tween-20, membrane was blocked for one hour in Odyssey Blocking Buffer (Li-Cor, Lincoln, NE) at RT. Mouse anti-human VE-cadherin (Cell Signaling Technology, Danvers, MA) (1:1000), mouse anti-human eNOS (BD Biosciences, San Jose, CA) (1:1000), rabbit anti-human VCAM-1 (Santa Cruz, Dallas, TX) (1:1000), rabbit anti-human NFκB (Cell Signaling Technology, Danvers, MA) (1:1000) and mouse anti-human GAPDH (Invitrogen, Grand Island, NY) (1:20,000) were used in Odyssey blocking buffer+0.1%Tween-20 to detect protein expression. The membrane was washed 4x in PBS-tween, 1x in PBS, and incubated overnight with primary antibody at 4°C with gentle agitation. The same washes were then performed and the membrane was incubated in Odyssey blocking buffer +0.1% Tween-20 +0.2% SDS with 1:20,000 anti-mouse (680nm) and anti-rabbit secondary antibodies (800nm) (Li-Cor IRDye, Lincoln, NE). Blots were imaged using the Odyssey Infrared system (Li-Cor, Lincoln, NE).

**Quantitative real-time polymerase chain reaction**

Total RNA was extracted from lysed 3-D hydrogels using a RNeasy total RNA purification kit (Qiagen, Valencia, CA) and quantified using a Nanodrop 2000 spectrophotometer (Nanodrop, Wilmington, DE). An equal amount of RNA from each sample was reverse transcribed to cDNA using the iScript™ cDNA synthesis kit (Bio-Rad, Hercules, CA). Quantitative real-time polymerase chain reaction was performed on all samples using SYBR Green PCR master mix (Applied Biosystems, Foster City, CA), and a CFX96 or MiniOpticon Real-Time PCR Detection System (Bio-Rad, Hercules, CA). Samples amplifying at > 37 cycles were considered non-detectable.

| **Primer Name** | **Forward** | **Reverse** |
| --- | --- | --- |
| ALP, *sus scrofa* | ATGAGCTCAACCGGAACA | GTGCCCATGGTCAATCCT |
| MMP-9, *sus scrofa* | ACACACACGACATCTTCC | AAGGTCACGTAGCCCACAA |
| MMP-2 | CGCCCATCATCAAGTTTC | TCGAGTTCGCCTGTCTG |
| Sox9 | GGAGACTGCTGAATGAGAGC | CGTTCTTCACCGACTTTCTC |
| Msx2 | AAGGCAATGACTTGTTTTCG | AGGCTGGAGACCTTGACG |
| COL1A1 | AGAAGACATCCCACCAGTCA | CGTCATCGCACAACACATTG |
| COL2A1 | GTCTACCCCAATCCAGCAAA | GTCTACCCCAATCCAGCAAA |
| COL3A1 | TTGGCCCTGTTTGCTTTTTA | TGGTTGACAAGATGAGAACAAAA |

***Ex vivo* experiments**

***Ex vivo* protein isolation**

Fresh, healthy, adult pig hearts were donated by Shirk Meats of Dundee, NY. Leaflets were removed carefully from the heart and transferred immediately to ice-cold sterile PBS. In less than one hour, sterile tools were used to cut leaflets in half and transfer each half to a single well of a 24-well plate, with 0.5mL of control or treatment media in each well. Treatments were designed with identical formulations as the *in vitro* experiments. Leaflet halves were paired across control and treatment conditions to increase robustness of comparison. Six or more samples were used for each individual treatment. Plates were cultured at 37^o^C and 5% CO_2_. 30ng/mL of TNFα was added directly to the media, supplemented with 10 μmol/L BH_4_ or 200U/mL Cu-Zn-Superoxide dismutase-polyethylene glycol, where noted. This culture media was changed every 48 hours for 21 days. TUNEL assay for apoptosis was performed to confirm cell viability, DHE was used to detect superoxide, Russell-Movat pentachrome staining was used to visualize extracellular matrix components and Alizarin Red S in combination with von Kossa staining was used to assess calcification, as describe below.

For *ex vivo* protein isolation, leaflet halves were transferred to a sterile mortar, flash frozen with liquid nitrogen, and crushed to a fine powder with sterile pestle. Mortar and pestle were washed and sterilized between each sample. Sample powder was transferred to 100 μL of 2x Laemmli buffer, heated to 70^o^C for 10 minutes, sonicated on ice, and spun down at 14,000 rpm for 10 minutes at 4^o^C. The supernatant was transferred to a new tube, flash frozen, and kept at -80^o^C until protein quantification. Protein was quantified as described for *in vitro* experiments and western blot performed as above.

***Ex vivo* RNA isolation**

Aortic valve leaflet tissue was flash-frozen and ground to a powder, similar to protein isolation procedure. Powder was transferred to eppendorf tube and vortexed with 350 μL of RLT lysis buffer (Qiagen, Hilden, Germany) +1% β-mercaptoethanol to mix. Any remaining un-dissolved tissue was homogenized using the QIAshredder system (Qiagen, Hilden, Germany). RNA solutions were flash frozen and stored at -80^o^C.

***Ex vivo* Histology & Immunofluorescence**

Aortic valve leaflet samples were fixed in 4% PFA overnight at 4^o^C, and then stored in ethanol until paraffin embedding. Samples were embedded with root to free-edge view exposed. Embedded sections were sectioned in 6μm increments and mounted on glass slides. Representative sections from each sample were stained with Alizarin Red S (VWR), von Kossa, or Russell-Movat pentachrome stain (Mastertech, Lodi, CA). For ARS, cells were deparaffinized, hydrated, incubated in ARS stain for 2 minutes, rinsed in xylene and xylene-acetone (1 min each), dehydrated in 3 changes of xylene (1 min each), and mounted. Analysis of ARS was done in ImageJ, using thresholding to identify boundaries of the leaflet section. Leaflet section with background subtracted was measured for area and integrated density of ARS stain. Similar deparaffinization and hydration was performed for von Kossa, followed by a 20 minute incubation in 1% aqueous silver nitrate under UV light. Slides were rinsed in water followed by 5% sodium thiosulfate (5 min), rinsed, and counter-stained with 0.1% nuclear fast red (5 min). Dehydration and mounting performed as above. Slides were imaged and thresholded in ImageJ so that only black-brown mineral deposits were selected for analysis. Mineral deposit area was measured and normalized to valve leaflet area. Russell-Movat pentachrome stain was performed according to manufacturer’s instructions, with the following adjustments: 30 seconds in 2% ferric chloride, 20 minutes in 1% alcian blue, and 1 minute in each change of 5% phosphotungstic acid. A color deconvolution algorithm [5] was used to separate individual dyes from the Russell-Movat stained sections into single-channel images. MATLAB© was used to find the integrated density of each single-channel image. Integrated density of each component (GAG = blue, collagen = yellow, elastin = black) was normalized to the area of the valve leaflet section being examined. The resulting output was divided by the average integrated density of each dye in control samples, producing fold change expression of GAG, elastin, and collagen relative to control.

Additional sections were immunofluorescently labeled as described above, with an additional antigen retrieval step in which slides were submerged in 98^o^C sodium citrate buffer (10 mM Sodium citrate, 0.05% Tween 20, pH 6.0) for five minutes. Immunofluorescently labeled slides were imaged with a Zeiss 710 laser scanning confocal microscope, as described in the *in vitro* methods. TUNEL assay (Invitrogen, Grand Island, NY was performed according to manufacturer’s instructions to label apoptosis, necrosis, and cell nuclei. Leaflet sections were imaged imaged with a Zeiss 710 laser scanning confocal microscope, analyzed, and quantified using ImageJ. Number of cell nuclei and cells positive for apoptosis per section were counted using particle analysis. Percent of cells positive for apoptosis (TUNEL stain) was presented.

**Statistical Analysis**

Data is expressed as mean +/- standard error of the mean (SEM). All comparisons between two groups were made using two-tailed, unpaired t-tests assuming unequal variance. Comparisons between multiple groups were made using ANOVA with Tukey post hoc paired tests. Differences between means were considered significant when p < 0.05.

**References**

1. Butcher JT, Penrod AM, Garcia AJ, Nerem RM. (2004) Unique Morphology and Focal Adhesion Development of Valvular Endothelial Cells in Static and Fluid Flow Environments. Arterioscler Thromb Vasc Biol 24:1429–34.

2. Skold BH, Getty R, Ramsey FK. (1966) Spontaneous atherosclerosis in the arterial system of aging swine. Am J Vet Res 27:257-73.

3. Holliday CJ, Ankeny RF, Nerem RM. (2011) Discovery of Shear- and Side-specific mRNAs and miRNAs in Human Aortic Valvular Endothelial Cells. Am J Physiol-Heart C 301:H856-67.

4. Miller JD, Chu Y, Brooks RM, Richenbacher WE, Pena-Silva R, et al. (2008) Dysregulation of Antioxidant Mechanisms Contributes to Increased Oxidative Stress in Calcific Aortic Valvular Stenosis in Humans. J Am Coll Cardiol 52:843–850.

5. Ruifrok AC, Johnston DA. (2001) Quantification of histochemical staining by color deconvolution. Anal Quant Cytol Histol 23:291-9.
